# Amendment with Burkina Faso phosphate rock-enriched composts alters soil chemical properties and microbial structure, and enhances sorghum agronomic performance

**DOI:** 10.1038/s41598-022-18318-1

**Published:** 2022-08-17

**Authors:** Adama Sagnon, Shinya Iwasaki, Ezechiel Bionimian Tibiri, Nongma Armel Zongo, Emmanuel Compaore, Isidore Juste O. Bonkoungou, Satoshi Nakamura, Mamoudou Traore, Nicolas Barro, Fidele Tiendrebeogo, Papa Saliou Sarr

**Affiliations:** 1 Laboratory of Molecular Biology, Epidemiology and Monitoring of Bacteria and Virus Transmitted by Food (LaBESTA), University Joseph KI-ZERBO, 03 BP 7021 Ouagadougou 03, Burkina Faso; 2 Laboratory of Virology and Plant Biotechnology, Institute of Environment and Agricultural Research (INERA), 01 BP 476 Ouagadougou 01, Burkina Faso; 3 Rural Development Division, Japan International Research Center for Agricultural Sciences, Tsukuba, 305-8686 Japan; 4 Department of Natural Resources Management and Production Systems, Institute of Environment and Agricultural Research (INERA), 01 BP 476 Ouagadougou 01, Burkina Faso; 5 Crop, Livestock and Environment Division, Japan International Research Center for Agricultural Sciences, Tsukuba, 305-8686 Japan

**Keywords:** Soil microbiology, Biogeochemistry, Geochemistry

## Abstract

Low soil available phosphorus (P) severely limits crop production in sub-Saharan Africa. The present study evaluated phosphate rock-enriched composts as locally available low-cost fertilizers for sorghum production. The treatments consisted of sorghum straw, compost (COMP), phosphate rock (BPR), BPR-enriched compost (P-COMP), BPR-rhizosphere soil-enriched compost (P-COMP-SOIL), nitrogen-phosphorus-potassium treatment (NPK, 60–39–25), and control (NK, 60–25). Sorghum straw and compost were applied at 1.34 tons ha^−1^. N, P, and K in all treatments, excluding the control, were adjusted to 60, 39, and 25 kg ha^−1^, with urea, BPR, and KCl, respectively. Sorghum vr. *kapelga* was cultivated and soil samples were collected at the S5, S8, and S9 growth stages. P-COMP-SOIL and NPK yielded better sorghum yields than the other treatments. The rhizosphere soil of P-COMP-SOIL had high abundance of soil bacteria and AMF, and genes involved in P solubilization, such as: acid phosphatase (*aphA*), phosphonatase (*phnX*), glucose dehydrogenase (*gcd*), pyrroloquinoline quinone (*pqqE*), phosphate-specific transporter (*pstS*). The superior performance of the P-COMP-SOIL was associated with its higher available P content and microbial abundance. Multivariate analysis also revealed vital contributions of N, carbon, and exchangeable cations to sorghum growth. Soils could be amended with phosphate rock-rhizosphere soil-enriched composts, as an alternative to chemical fertilizers.

## Introduction

Sub-Saharan Africa is experiencing significant population growth, which is expected to increase from 1.03 billion in 2017 to 3.08 billion in 2100^1^, and demands increased agricultural production for food security, a key sustainable development goal. However, several factors constrain sustainable crop production in the region, including poor levels of essential soil nutrients, particularly phosphorus (P)^2^. Although chemical fertilizer application is a strategy used extensively to improve soil nutritional quality and agricultural yield, the low financial returns of farmer communities in sub-Saharan Africa hinder their adoption^3^. In regions where chemical fertilizers are applied excessively, there is an increasing awareness of their negative effects on soil biological properties and the ecological environmental, in addition to lower agricultural yields over the long term^4^. Furthermore, excessive nitrogen (N) fertilizer use contributes to global warming because of the release of greenhouse gases, such as potent nitrous oxide (N_2_O), whereas excessive P fertilizer input can lead to eutrophication^5^.

Therefore, it is necessary to explore innovative fertilizers that do not harm the environment, for example, in the form of pollution of eutrophication, which hinder sustainable agriculture. Easily accessible resources, such as agricultural residue, livestock by-products, and locally sourced phosphate rocks, have been considered alternative soil inputs that could improve soil properties and enhance plant growth and productivity^6^. They could improve the soil nutrient budget, shape soil microbial populations^7^, and protect plants from pathogens^8^. However, the amounts of nutrients available in compost are often too inadequate to meet the nutritional needs of plants. Phosphate rock (PR), a material existing in large deposits in several African countries, including Burkina Faso, is a soil fertilization strategy based on direct application or amendment to compost, which improves compost P content.

Sub-Saharan African PRs can achieve high performance in lowland rice following direct application, regardless of PR reactivity or location^9^. However, under water-limited conditions, such as upland cultivation of rice or other crops, the effects of the initial direct PR application may be depressed, although it may enhance soil total P, with residual effects in subsequent cultivations^10,11^. Because smallholder farmers are keener on high yields in the first year of cultivation than in subsequent cultivations, it is essential to improve the agronomic effectiveness of PR. PR has been suggested as a phosphate supplement for obtaining compost rich in plant-available P^12^. In addition, the available P pool in PR-enriched compost may be increased by adding rhizosphere soil as a niche for beneficial microbes during the composting process, increasing the abundance of phosphate-solubilizing fungi and the microbial alkaline phosphatase gene, which participates in organic P mineralization^13^. Phosphate-solubilizing microorganisms (PSM) are essential for phosphate availability from organic and inorganic compounds in the soil^14,15^. These include bacteria, actinomycetes, fungi, and some algae^16^, which use various mechanisms to make phosphate available to plants. Rhizosphere acidification and phosphatase production play crucial roles in inorganic P solubilization and organic P mineralization^15^. Determining the diversity and abundance of PSM genes in the environment (soil, organic, and inorganic fertilizers) is essential for the understanding of their potential contributions to P bioavailability. However, whether the P pool made available to plants by PSMs is considerably enough to translate into increased crop yield remains controversial. According to some researchers, PSMs do not improve crop P nutrition in complex soil–plant systems^17^, whereas many other studies^18,19^ generally performed under controlled conditions have demonstrated increases in crop yield. The discrepancy observed in the effect of PSMs requires further investigation to elucidate the role of PSMs in crop production under field conditions subjected to several biotic and abiotic factors.

The present field study evaluated the agronomic performance of organic material composed of sorghum straw residues and Burkina Faso phosphate rock (BPR)-enriched composts, which has been previously described^13^, when compared with the direct application of BPR and NPK chemical fertilizers. Their effects on sorghum (*Sorghum bicolor* [L.] Moench) growth and grain production were also assessed. Although the low available P in soil constrains sorghum production in West and Central Africa, it is still cultivated widely in the region, primarily between the 500-and 100-mm rainfall isohyets, and is a staple food for a large proportion of the population^20^. The effects of such amendments on soil biological and chemical properties were also investigated.

Biological analyses have focused on the rhizosphere area, which are directly influenced by plant roots and exudates^21,22^. Root exudates and rhizodeposition make the rhizosphere microenvironment a hotspot for microbial growth and activity^23^, with beneficial interactions with plants through diverse molecular and chemical processes that facilitate water and nutrient uptake^24^. We quantified the abundance of the bacterial inorganic phosphate-solubilizing glucose dehydrogenase (*gcd*) gene^25,26^ and pyrroloquinoline quinone (*pqq*) gene, which encodes the cofactor pyrroloquinoline quinone (PQQ)^27^. Glucose dehydrogenase (GDH), an enzyme produced by activation of the gcd gene, is crucial for oxidization of glucose into gluconic acid, an organic acid that solubilizes inorganic P.

## Materials and methods

### Study site

The field experiment was conducted at the Environmental and Agricultural Research and Training Center (CREAF) in Saria, (12°16’N, 2°09’W, 300-m altitude) in the central west region of Burkina Faso. The soil contained 0.25 g kg^−1^ dry soil total N (TN); 2.46 g kg^−1^ dry soil total carbon (C; TC); a C/N ratio of 9.69; 5.78 mg Bray1-P kg^−1^ dry soil; 5.28 mg Bray2-P kg^−1^ dry soil; 2.91, 0.01, 0.07, 0.62, 0.25, and 0.94 cmolc kg^−1^ dry soil of CEC, Na, K, Ca, Mg, and the sum of exchangeable cations, and a pH (H_2_O) of 5.07. It is a Lixisol^28^ characterized by low soil fertility, low water-holding capacity, and a soil surface crust that causes low water infiltration^29^. The experiment was conducted during the rainy season from July to November 2019. The area received 911.9 mm of rain on 70 rainy days during the 2019 cropping season, according to meteorological data obtained from the CREAF/Saria station. The average monthly air temperature, relative humidity, air pressure, and wind speed from July to November were 26.80 °C, 78.68%, 976.25 hPa, and 0.98 m/s, respectively. The wind speed was relatively higher in July and August, with an average of 1.25 m/s, and the relative humidity was slightly higher during the July–September period with an average of 83.01%. During the cropping season, the soil volumetric water content in the 0–10 cm and 10–25 cm soil layers was higher in July, August, and early September than in the other months, with a peak in August, when the area received the highest amount of rain^30^.

### Experimental design and sorghum cultivation

Three compost types, consisting of compost exclusively (COMP), BPR-enriched compost (P-COMP), BPR, and rhizosphere soil-enriched compost (P-COMP-SOIL), were prepared as described in an earlier report^13^. Briefly, Comp was prepared from sorghum straw residue (100 kg oven-dry basis). P-Comp comprised sorghum straw and 10% BPR. For P-Comp-Soil, 10% BPR and 10% rhizosphere soil were added to the sorghum straw. Rhizosphere soil was collected from a sorghum field by uprooting some plants and gently shaking them to remove loose soil. The remaining soil that adhered to the roots was shaken relatively vigorously, and the soil recovered from several sorghum stands was collected and considered rhizosphere soil. Notably, rhizosphere soil is defined differently in several studies, although it is generally accepted to be the soil tightly adhering to soil roots at approximately 1 mm. The rhizosphere soil used here for composting may include some non-rhizosphere soil from the vicinity of the roots, since relatively high soil amounts were required for the experiment. The soil was transported to the laboratory, stored under cold conditions, and used for composting three days after collection. It contained initial P and N concentrations of 1.15 g and 1.00 g N kg^−1^ dry soil, respectively. Each compost type was prepared in triplicate on blue sheets containing 100 kg of composting material. The C/N ratio of sorghum straw was adjusted to 25/1 with urea to facilitate decomposition. The compost piles were turned every two weeks, and the moisture content was adjusted to 65% throughout the composting period. At compost maturity (six months), total P, available P, total N, and pH were assessed, and the obtained data were used to calculate the amount of compost for application in the sorghum field experiments.

The influence of the three compost types and four other treatments, consisting a negative control with only N and K (NK), sorghum straw (SS), BPR, and NPK, yielding seven treatments, on sorghum growth and soil chemical and biological properties, was assessed in the present study. Urea provided N, K was supplied by KCl, and P was supplied by Triple Super Phosphate (TSP), in the NK and NPK treatments. In the experiment, similar amounts of N (60 kg ha^−1^) and P (39 kg ha^−1^) were applied in all treatments, excluding the negative control, which had no P application. Since K is not limited in the soil, KCl was uniformly applied in all treatments at a rate of 25 kg K ha^−1^, although the organic fertilizers may add slightly more levels of K. We calculated the amount of fresh organic material for field application according to the total P concentration in the composts and sorghum straw (Table S1). As a result, 146, 44, 1.34, and 1.54 t ha^−1^ would be required to apply 39 kg P ha^−1^ (90 kg P_2_O_5_ ha^−1^) using the sorghum, compost, P-compost, and P-compost-soil treatments, respectively. Therefore, we selected the lowest application rate of 1.34 t ha^−1^ organic material. Where necessary, we added BPR to leverage the total P at 39 kg ha^−1^. After applying 1.34 t ha^−1^ of organic material, the amount of total N was also adjusted to 60 kg ha^−1^ by adding urea (Table S1). The lower the amount of P applied, the lower the amount of organic material. For example, if 15 kg P ha^−1^ were applied, as is often the case in the area, only 0.5 t ha^−1^ of organic material would be required, and it would be challenging to spread such an amount in the plot area (6 m × 4 m). We arbitrarily applied a high rate of 39 kg P ha^−1^ (90 kg P_2_O_5_ ha^−1^) to an adequate amount of organic material (sorghum straw, Compost, P-compost, and P-compost-soil). Table S2 shows the adjustments in the amounts of P, N, and K in the different treatments. In the present study, all the treatments, excluding NK, supplied the same amount of total P. However, TSP and P-COMP-SOIL contained higher labile-P than the other treatments. BPR contained 12.05% P, corresponding to 36.87% PO_4_^31^.

The experimental design was a completely randomized Fisher block design with the seven treatments described above, with five replicates in each treatment. After plowing, the fertilizers were spread in the corresponding plots (4 × 6 m), which were 1.5 m apart inside and between blocks. The sorghum variety *kapelga* was sown with 80 cm × 40 cm spacing on July 16, 2019, followed by reseeding of the non-germinated hills on July 26, 2019. Each plot contained 75 hills, and the seedlings were thinned to two individual plants per hill. Weeds were regularly removed and insect attacks were controlled during the cultivation period. Rain was the sole source of water, and there was no irrigation.

In an area next to the sorghum cultivation experiment located at a distance of 100 m, two other plots were set up to assess changes in available P in soil when P fertilizers were applied with no plant cultivation. P (50 kg P ha^−1^) as TSP (156 kg ha^−1^) were uniformly broadcast in plots, and three composite soil samples were collected immediately per plot at 0–10 cm soil depth. The plots were left uncultivated and were weeded regularly. The second soil sampling was conducted during the harvest period. As described below, available soil P was determined after extraction, using the Bray2 method.

### Soil sampling

Bulk and rhizosphere soil samples were collected 52 days after sowing (DAS), 93 DAS, and 115 DAS, corresponding to the S5 (boot), S8 (hard dough), and S9 (physiological maturity) growth stages of the sorghum *kapelga* variety, to characterize changes in soil properties during the vegetative (S5) and grain filling (S8, S9) periods. Three random sorghum stands at the borders were uprooted and shaken vigorously to remove the loose soil. The rhizosphere soil adhering to the roots and under the influence of exudates (approximately 100 g plot^-1^) was meticulously recovered in clean containers while avoiding contamination between plots and between soil and substances from roots. The zone influenced by root exudates varies according to plant type, root system, and growth period; consequently, a defined method for extracting rhizosphere soil is lacking. However, numerous reports suggest dipping the root system into phosphate-buffered saline and recovering the rhizosphere soil following centrifugation^32^, whereas others use brushes and forceps. After collecting the rhizosphere soil, approximately 20 g per sample was put in 50-ml falcon tubes, immediately stored in an icebox, and transported to the laboratory within three hours. Samples were stored at − 20 °C until DNA extraction and molecular analysis. The remaining rhizosphere soil was air-dried for chemical analysis. In addition, composite bulk soil samples were collected from the inter-rows of each plot at a 0–10 cm soil depth using a core soil sampler for chemical analysis.

### Harvesting of sorghum plants

Plants were harvested at 115 DAS from an area delimited at the center of the plot containing 21 plant hills. The biomass was first air-dried for two weeks under the sun. The air-dried biomass in each plot was weighed, and a sub-sample was oven-dried at 75 °C for 48 h for use in calculating the moisture content and determining dry matter per plot. At harvest, air-dried grain weights were recorded after dehulling panicles. In addition, oven-dried grain yields were calculated in the same way as the biomass. Finally, all the dry yields were converted to t ha^-1^. After counting the number of panicles per yield area, the percentage of panicles filled with grain was calculated, as an indicator of crop production.

### Soil chemical analysis

The air-dried soil samples were sieved with a 2-mm mesh for chemical analysis at the Soil–Plant Laboratory of the Japan International Research Center for Agricultural Sciences (JIRCAS), Tsukuba, Japan. The pH was measured in a 1:2 soil: distilled water slurry using a compact pH meter LAQUAtwin-pH-22 (Horiba Scientific, Japan). TC and TN were determined using the dry combustion method using an NC analyzer (Sumigraph NC-220; Sumika Chemical Analysis Service, Ltd., Japan). Exchangeable cations were extracted using 1 M ammonium acetate solution with a pH 7.0^33^. The cation concentrations were determined using Inductively Coupled Plasma Optical Emission Spectroscopy (ICP-OES) using an ICPE-9000 (Shimadzu Inc. Tokyo, Japan). Soil available P was extracted using Bray1 (initial soil only) and Bray2 extracting solutions^34^, and the concentrations of P in the filtrate were determined using the colorimetric method^35^ with a UV-1800 spectrophotometer (Shimadzu, Japan).

### Soil DNA extraction

Rhizosphere soil stored at − 20 °C was left at in the room (25 °C) for approximately one hour. Subsequently, 0.25 g per sample was used for total DNA extraction using the DNeasy PowerSoil Pro Kit (QIAGEN, Germany) according to the manufacturer's instructions, with slight modification. In step five (5), we used a TissueLyser II **(**QIAGEN, Germany) at maximum speed for 5 min instead of a rotary shaker. After assessing the concentration using a Qubit HS fluorometer, the DNA was used for qPCR. Prior to that, soil moisture content was determined using the following formula by placing approximately 0.5 g of fresh soil on a hot plate at 100 °C for three (3) hours:

$$\% {\text{ moisture }} = \frac{fresh\, soil\, weight - dry\, soil\, weight}{{fresh\, soil\, weight}} \times 100$$

### Determination of the abundance of rhizosphere soil microbial genes

Rhizosphere soil DNA was used to quantify the abundance of total bacteria (16S rRNA), fungi (ITS), arbuscular mycorrhizal fungi (AMF), and several genes involved in phosphate solubilization. The detailed methods have been described in an earlier report^13^. P-solubilizing genes include glucose dehydrogenase (*gcd*)^25^ and pyrroloquinoline quinone (*pqqE*)^27^, as a cofactor of *gcd* that synthesizes organic acids responsible for the solubilization of inorganic phosphate. Other analyzed genes are involved in the mineralization of organic phosphates, such as acid phosphatase (*aphA*), alkaline phosphatase (*phoD*), phosphonatase (*phnX*), enterobactin-mediating siderophore (*entA*), and phosphate-specific transporter (*pstS*)^36^. AMF were amplified using AML1 (5’-ATCAACTTTCGATGGTAGGATAGA-3’) and AML2 (5’- GAACCCAAACACTTTGGTTTCC-3’) primers^37^, with the annealing temperature set to 58 °C for both PCR and qPCR. The amplification efficiencies/r^2^ values are summarized in Supplementary Table S3.

### Statistical analysis

The chemical and microbiological properties of the rhizosphere soil and the chemical properties of the bulk soil were subjected to two-way Analysis of Variance (ANOVA), using CropStat ver. 7.2 software (IRRI, Philippines) to determine the interactions between the sampling periods and treatments. In the absence of a significant interaction, the average values of the treatments for the three sampling periods were considered (n = 9). Data from each sampling period were analyzed separately using one-way ANOVA, if the interaction was significant (*p* < 0.05). When significant effects were observed, mean differences were compared using Fisher’s LSD (least significant difference (LSD) or Duncan’s multiple range test (DMRT) only if the number of treatments was equal to or greater than six.

Nonmetric multidimensional scaling (NMDS) was performed using the VEGAN package in R version 4.0.0^38^ to study the general relationships between sorghum yield components (dry grain and biomass yields) and the chemical and microbiological properties of the analyzed rhizosphere soil during the three sampling periods. Before running the NMDS, the average data for five replicates per variable were standardized. NMDS (with the highest scores) was visualized based on NMDS1/NMDS2 using the ggplot2 package in R^39^. Spearman’s rank correlations (rs) between yield components and soil properties were obtained using PAST v.2.17^40^.

### Ethical statement

This research, conducted jointly by researchers of the Japan International Research Center for Agricultural Sciences (JIRCAS) and the Environmental Institute for Agricultural Research (INERA) of Burkina Faso, included the collection of soil and plant samples from the field in Burkina Faso. Plant and soil samples and soil DNA were transferred from INERA (Burkina Faso) to JIRCAS (Japan) for chemical and biological analyses. The transfer of samples was performed under a joint research contract and material transfer agreement between the two institutions. Laboratory analyses followed all applicable institutional, national, and international guidelines and regulations.

## Results

### Sorghum yields

NPK treatment increased sorghum total biomass yield and grain yield significantly than all treatments, excluding the P-COMP-SOIL treatment (Table 1). The total biomass obtained from the P-COMP-SOIL treatment (3.87 t ha^-1^) was not significantly different from that of the control treatment (2.91 t ha^-1^), but was significantly higher than that of the other four treatments. The P-COMP-SOIL treatment had grain yields comparable to those of NK and P-COMP treatments, whereas the sorghum straw, COMP, and BPR produced minor sorghum grain yields. The remaining treatments had less influence on yield components. In addition, NPK and P-COMP-SOIL had the highest percentages of panicles filled with grains (72.5 and 72.2%, respectively).

**Table 1:** Effect of organic and organo-mineral amendments on sorghum growth properties following one-way Analysis of Variance (ANOVA).

Treatments	Dry biomass	Dry grains	% filled panicles
	t ha^−1^		
NK	2.91 bc	0.63 bc	55.03 c
Sorghum straw	2.34 c	0.48 c	57.66 bc
COMP	2.27 c	0.45 c	61.21 abc
P-COMP	2.59 c	0.61 bc	67.37 ab
P-COMP-SOIL	3.87 ab	0.82 ab	72.23 a
BPR	2.29 c	0.49 c	54.56 c
NPK	4.43 a	0.86 a	72.51 a
p rate	*	*	*
SE	0.49	0.09	5.01
Error df	24	24	24

Within the same column, values assigned with the same letter are not significantly different according to the Student–Newman–Keuls (SNK) test at the 5% probability level. Parameter values assigned to different letters differ significantly (*) at *p* < 0.05. *NK* Without phosphate or compost addition, *COMP* Sorghum straw-based compost, *P-COMP* Sorghum straw-based compost + BPR, *P-COMP-SOIL* Compost made from sorghum straw, BPR, and sorghum rhizosphere soil, *BPR* Burkina phosphate rock, *filled ears* Ears filled with grains (the percentage is calculated based on the total number of ears found in a harvest area and the number of ears filled with grain), *SE* Standard error, *Error df* Error degree of freedom.

### Soil chemical properties

Two-way ANOVA results showed no significant interaction between sampling period and treatments (data not shown) for TN, TC, and C/N (bulk soil), and for these elements plus the sum of exchangeable cations (rhizosphere soil). Therefore, Table 2 summarizes the mean values across the sampling periods and shows no variation in TN, TC, and C/N among treatments in bulk soil, but C/N was higher at harvest (S9) than at 52 DAS (S5). TN in the rhizosphere soil was significantly higher under the P-COMP-SOIL, P-COMP, and COMP treatments than under NK, sorghum straw, and BPR treatments. However, the TN values in the COMP and P-COMP treatments were similar to those in the NPK treatment. Rhizosphere soil collected from sorghum plants that received P-COMP-SOIL contained significantly higher concentrations of exchangeable cations (1.08 cmolc kg^−1^ dry soil) than the rhizosphere soil obtained from the control and BPR treatments. Generally, the rhizosphere soil had higher TN and TC values at 52 DAS (S5), which later decreased as cultivation progressed.

**Table 2:** Chemical properties of bulk and the rhizosphere soils following two-way Analysis of Variance (ANOVA).

Variables	TN		TC		C/N	Sum exch. cations
	Bulk	Rhiz	Bulk	Rhiz	Bulk	Rhiz
	g kg^−1^ dry soil					cmolc kg^−1^ dry soil
**Treatment’ effect (DMRT-based comparison)**						
NK	0.264	0.338 cd	2.451	2.976	9.32	0.876 c
Sorghum straw	0.282	0.331 d	2.476	3.089	8.89	0.938 b
COMP	0.29	0.355 ab	2.479	3.123	8.75	0.985 b
P-COMP	0.267	0.365 ab	2.538	3.212	9.52	0.963 b
P-COMP-SOIL	0.265	0.370 a	2.517	3.266	9.5	1.079 a
BPR	0.263	0.335 d	2.473	2.954	9.43	0.870 c
NPK	0.271	0.352 bc	2.45	3.127	9.09	0.949 b
p rate	ns	*	ns	ns	ns	*
SE	0.028	0.007	0.327	0.098	1.52	0.026
Error df	2	83	2	83	2	81
LSD_0.05_ (n = 15)	0.167	0.022	1.96	0.301	9.15	0.08
**Sampling period’s effect (LSD-based comparison)**						
52DAS (S5)	0.291	0.389 a	8.20 a	2.365	8.20 a	0.972
93DAS (S8)	0.254	0.336 b	9.24 ab	2.355	9.24 ab	0.899
115DAS (S9)	0.27	0.323 b	10.20 b	2.73	10.20 b	0.983
p rate	ns	**	*	ns	*	ns
LSD_0.05_ (n = 35)	0.063	0.034	1.47	0.846	1.47	0.16

Within the same column, values assigned the same letter are not significantly different according to the Student–Newman–Keuls (SNK) test at the 5% probability level. Values assigned different letters differ highly significantly (**) at *p* < 0.01, and significantly (*) at *p* < 0.05, *ns* Not significant at *p* < 0.05. *NK* Without phosphate or compost addition, *COMP* Sorghum straw-based compost, *P-COMP* Sorghum straw-based compost + BPR, *P-COMP-SOIL* Compost made from sorghum straw, BPR, and sorghum rhizosphere soil, *BPR* Burkina phosphate rock, *Rhiz* Rhizosphere, *DAS* Days after sowing, [*S5, S8, S9*] = stage 5 (boot), stage 8 (hard dough), and stage 9 (physiological maturity) of the sorghum variety *kapelga*, *SE* Standard error, *df* Error degree of freedom, *TC* Total carbon, *TN* Total nitrogen, *Exch. Cat.* Exchangeable cations (Ca, K, Mg, Na), *DMRT* Duncan Multiple Range Test, *LSD* Least significant difference.

The interactions between sampling periods and treatments were significant for pH, C/N, and Bray2-P in the rhizosphere soil and Bray2-P in the bulk soil (data not shown). One-way ANOVA results are presented in Table 3. Rhizosphere soil pH showed significant differences among treatments at S5 and S8. At S5, the pH was less acidic where organic matter (P-COMP-SOIL, P-COMP, COMP, Sorghum straw) was applied, whereas the BPR treatment had a higher pH (5.90) at S8. As shown in Table 3 and highlighted in Fig. S1, rhizosphere pH increased with the sorghum growth stage. The C/N ratio of the rhizosphere soil at 93 DAS was significantly higher (9.52) in the sorghum straw treatment than that in the other treatments. The pH and exchangeable cations of bulk soils were determined in samples collected during the sorghum growth period and showed no significant differences among treatments.

**Table 3:** Chemical parameters of bulk and rhizosphere soils over sampling periods following one-way Analysis of Variance (ANOVA).

Treatments	pH				C/N			SEC	Bray2-P (mg kg^−1^ dry soil)					
	52DAS	93DAS	115DAS		52DAS	93DAS	115DAS	115DAS	52DAS	93DAS	115DAS	52DAS	93DAS	115DAS
	Rhiz		Rhiz	Bulk	Rhiz			Bulk	Bulk			Rhiz		
**Treatment’s effect (DMRT-based comparison)**														
NK	5.34 c	5.53 bc	5.95	5.39	8.66	8.73 b	9.05	0.787	8.32	9.67 bc	7.23 d	09.39 e	08.01 e	09.71 c
Sorghum straw	5.49 ab	5.67 b	6.17	5.42	8.79	9.52 a	9.67	0.827	10.35	12.06 ab	12.36 c	28.49 ab	31.89 ab	35.50 a
COMP	5.48 ab	5.59 bc	6.09	5.44	8.77	8.36 b	9.24	0.915	13.21	13.82 a	20.18 b	19.97 d	34.20 a	34.03 a
P-COMP	5.53 ab	5.60 bc	5.89	5.47	8.75	8.56 b	9.09	0.873	7.24	5.93 d	9.93 cd	12.06 e	16.70 d	23.83 b
P-COMP-SOIL	5.60 a	5.64 b	5.93	5.58	8.97	8.55 b	8.85	0.91	9.86	8.07 cd	12.95 c	20.88 c	25.23 c	23.85 b
BPR	5.43 bc	5.90 a	5.67	5.37	8.75	8.32 b	9.32	0.786	10.28	14.03 a	12.36 c	26.98 b	30.32 abc	30.23 ab
NPK	5.15 d	5.46 c	5.79	5.83	8.73	8.35 b	9.53	0.861	14.59	14.67 a	28.20 a	32.81 a	26.27 bc	24.29 b
p rate	**	*	ns	ns	ns	**	ns	ns	ns	**	***	***	***	***
SE	0.055	0.072	0.11	0.06	0.14	0.216	0.185	0.068	1.76	1.68	2.13	1.905	2.723	3.353
Error df	24	24	24	1.18	24	24	24	0.198	24	24	24	24	24	24

Within the same column, values assigned the same letter are not significantly different according to the Student–Newman–Keuls (SNK) test at the 5% probability level. Values assigned different letters differ highly significantly (***) at *p* < 0.001, (**) at *p* < 0.01, and significantly (*) at *p* < 0.05, *ns* Not significant at *p* < 0.05. *NK* Without phosphate or compost addition, *COMP* Sorghum straw-based compost, *P-COMP* Sorghum straw-based compost + BPR, *P-COMP-SOIL* Compost made from sorghum straw, BPR, and sorghum rhizosphere soil, *BPR* Burkina phosphate rock, *Rhiz* Rhizosphere, *DAS* Days after sowing, *SE* Standard error, *Error df* Error degree of freedom, *SEC* Sum of exchangeable cations (Ca, K, Mg, Na) with the unit cmolc kg^−1^ dry soil, *DMRT* Duncan multiple range test.

Bray2-P was the most variable element among the analyzed soil chemical properties (Table 3). The bulk soil of the plots fertilized with COMP, BPR, and NPK had significantly higher Bray2-P at 93 DAS than that of the other treatments. However, Bray2-P was only higher in the bulk soil of the NPK-fertilized plots at 115 DAS. The Bray2-P content in the rhizosphere soil had a contrasting pattern, being significantly higher in the NPK (32.81 mg kg^−1^ dry soil) and Sorghum straw (28.49 mg kg^−1^ dry soil) treatments than in the other treatments, at 52 DAS. The control and P-Comp treatments recovered less Bray2-P during this period. At 93 DAS, the COMP, Sorghum straw, and BPR-treated soil had significantly higher P, with 34.20, 31.89, and 30.32 mg kg^−1^ dry soil, but available P levels in the sorghum Straw and BPR-treated soil were similar to that in the NPK treatment. The rhizosphere soils of the Sorghum straw, COMP, and BPR treatments had significantly higher P levels at 115 DAS, similar to that at 93 DAS. Overall, Bray2-P in the rhizosphere soil was higher than in the bulk soils (Table 3 and Fig. S2). Specifically, Bray2-P in the NPK treatments, where TSP was the P source, increased rapidly in the rhizosphere soil until 52 DAS and later decreased. In contrast, the increase was moderate in the bulk soil up to 93 DAS. Afterward, it increased considerably toward the physiological maturity stage. In addition, the bulk soils contained almost similar amounts of Bray2-P as the initial soil, excluding in the NPK and COMP treatments, until 93 DAS. Notably, Bray2-P, following TSP addition to bare soil, increased suddenly due to fertilization. However, it decreased drastically after four months (Fig. S3), although no plant was cultivated, which is consistent with the lower P availability in the bulk soil than in the rhizosphere soil in the field experiment. In addition, rhizosphere soils had higher TN, TC, and exchangeable cations than bulk soils (Tables 2 and 3).

### Microbial gene abundance in rhizosphere soil

Microbiological analysis in the present study focused on the rhizosphere soil, which is influenced by roots. Two-way ANOVA results showed significant interactive effects (data not shown) between sampling periods and treatments on the abundance of *gcd*, AMF, and *pstS* genes. The mean values obtained by one-way ANOVA are presented in Table 4. Significant differences in *gcd* gene abundance among treatments were observed only at 52 DAS, where the P-COMP-SOIL treatment contained a significantly higher abundance in the *gcd* gene than the other treatments. Conversely, the NPK treatment had a significantly lower abundance in the *gcd* gene than NK.

**Table 4:** One-way ANOVA (treatment) of *gcd*, AMF, *pstS* genes over the three sampling periods.

Treatments	*gcd*			AMF			*pstS*		
	52DAS	93DAS	115DAS	52DAS	93DAS	115DAS	52DAS	93DAS	115DAS
NK	3.20E+05 b	2.50E+05	2.94E+05	6.75E+05 d	8.12E+05	2.42E+06 cd	3.93E+05 b	7.47E+05	5.57E+05 b
Sorghum straw	2.60E+05 bc	3.20E+05	3.03E+05	9.41E+05 bcd	9.76E+05	7.30E+06 a	4.40E+05 b	4.61E+05	6.19E+05 b
COMP	2.91E+05 bc	3.55E+05	2.20E+05	9.99E+05 abc	1.01E+05	4.05E+06 b	6.07E+05 b	4.38E+05	6.90E+05 b
P-COMP	2.64E+05 bc	2.38E+05	3.19E+05	1.27E+06 a	8.81E+05	3.69E+06 bc	5.92E+05 b	3.75E+05	1.03E+06 a
P-COMP-SOIL	4.74E+05 a	2.87E+05	2.59E+05	1.13E+06 ab	1.22E+06	2.64E+06 cd	1.26E+06 a	4.93E+05	5.90E+05 b
BPR	2.92E+05 bc	3.29E+05	2.80E+05	9.48E+05 bcd	1.23E+06	2.31E+06 d	9.86E+05 a	5.86E+05	5.23E+05 b
NPK	2.10E+05 c	2.59E+05	2.64E+05	7.44E+05 cd	1.38E+06	2.58E+06 cd	5.50E+05 b	5.95E+05	6.93E+05 b
p rate	**	ns	ns	*	ns	***	***	ns	*
SE	3.79E+04	3.82E+04	3.11E+04	1.29E+05	2.21E+05	5.88E+05	1.27E+05	7.97E+05	1.08E+05
Error df	24	24	24	24	24	24	24	24	24

*NK* Without phosphate or compost addition, *COMP* Sorghum straw-based compost, *P-COMP* Sorghum straw-based compost + BPR, *P-COMP-SOIL* Compost made from sorghum straw, BPR, and sorghum rhizosphere soil, *BPR* Burkina phosphate rock, parameter values assigned by different letters differ significantly (**) at *p* < 0.01 and significantly (*) at *p* < 0.05, *ns* Not significant at *p* < 0.05. *SE* Standard error, *df* Error degree of freedom, *DMRT* Duncan’s multiple range test, *LSD* least small difference, *gcd* Glucose dehydrogenase, *AMF* Arbuscular mycorrhizal fungi, *pstS* Phosphate-specific transporter, *DAS* Days after sowing.

At 52 DAS, the AMF abundance was higher in the rhizosphere soil of the P-COMP treatment than in NK, sorghum straw, BPR, and NPK treatments. We observed no significant differences in AMF abundance among the P-COM, P-COMP-SOIL, and COMP treatments. In contrast, AMFs were significantly higher under sorghum straw than under the other treatments at 115 DAS. At 115 DAS, the rhizosphere soil of NK, P-COMP-SOIL, BPR, and NPK treatments had significantly fewer AMF copy numbers than sorghum straw and P-COMP. Moreover, the AMF population at 115 DAS was three-fold that at 52 DAS. *pstS* gene abundance differed among treatments at 52 and 115 DAS, similar to that of AMF. It was significantly higher in the P-COMP-SOIL and BPR treatments at 52 DAS than in the other five treatments. At 115 DAS, the P-COMP had significantly higher *pstS* gene abundance than the other treatments.

Table 5 presents the mean values of the three sampling periods for the remaining genes (*phnX*, *phoD*, *ITS fungi*, *16S rRNA*, *entA, pqqE*, and *aphA*), with significant interactions between the sampling periods and treatments. Significant effects (*p* < 0.05) on *phnX, ITS, 16S rRNA*, and *aphA* gene abundances, and a highly significant effect (*p* < 0.01) on *pqqE* gene abundance, were observed. COMP, P-COMP, and P-COMP-SOIL promoted the proliferation of microorganisms harboring the *phnX* gene more significantly than the other treatments. Soil fungi (*ITS*) were significantly more abundant under the NPK (4.85 10^7^ copies g^−1^ dry soil) treatment than the other treatments. The NK-amended soil had the lowest fungi abundance (3.37 10^7^ copies g^−1^ dry soil), which was not significantly different from that in the sorghum straw treatment. The P-COMP-SOIL treatment enhanced the population of soil bacteria significantly (5.17 10^8^ copies of *16S rRNA* g^−1^ dry soil) when compared to the NK, BPR, and NPK treatments; however, there were no significant differences with the sorghum straw, COMP, and P-COMP treatments. The *pqqE*, a cofactor of the *gcd* gene involved in inorganic phosphate solubilization, was significantly higher under the COMP, P-COMP-SOIL, and NPK treatments, whereas the BPR treatment had the lowest *pqqE* abundance. In contrast, the P-COMP-SOIL treatment significantly enhanced the copy numbers of *aphA* (4.98 10^5^ copies g^−1^ dry soil), followed by the P-COMP treatment, with 2.91 10^5^ copies g^−1^ dry soil.

**Table 5:** Abundance of some microbial genes in the rhizosphere of sorghum.

Variables	*phnX*	*phoD*	*ITS* fungi	*16S rRNA*	*entA*	*pqqE*	*aphA*
	Copy number (g^−1^ dry soil)						
**Treatment effect (comparison based on DMRT)**							
NK	3.45E+05 b	9.56E+06	3.37E+07 d	4.38E+08 bc	3.61E+06	3.53E+05 b	1.69E+05 c
Sorghum straw	3.60E+05 b	1.06E+07	3.63E+07 cd	4.61E+08 ab	4.49E+06	3.94E+05 b	2.13E+05 bc
COMP	4.42E+05 a	1.04E+07	4.23E+07 b	4.56E+08 ab	3.60E+06	8.19E+05 a	1.37E+07 c
P-COMP	4.12E+05 a	9.06E+06	3.60E+07 cd	4.89E+08 ab	3.64E+06	2.88E+05 bc	2.91E+05 b
P-COMP-SOIL	4.22E+05 a	1.09E+07	4.08E+07 b	5.17E+08 a	3.96E+06	7.93E+05 a	4.98E+05 a
BPR	3.59E+05 b	8.52E+06	3.95E+07 bc	3.88E+08 c	4.04E+06	2.03E+05 c	1.91E+05 c
NPK	2.93E+05 c	1.01E+07	4.85E+07 a	4.39E+08 bc	3.42E+06	8.43E+05 a	1.53E+05 c
p rate	*	ns	*	*	ns	**	*
SE	2.13E+04	8.02E+05	1.78E+06	2.68E+07	4.63E+05	5.07E+04	3.75E+04
Error df	52	56	51	49	50	43	29
**Effect of sampling period (comparison based on LSD)**							
52DAS (S5)	2.88E+05 b	1.22E+07 a	3.30E+07	4.35E+08	3.95E+06 a	1.13E+06 a	3.11E+05
93DAS (S8)	3.23E+05 b	9.44E+06 b	4.10E+07	4.36E+08	2.82E+06 b	2.71E+05 b	2.55E+05
115DAS (S9)	5.18E+05 a	8.05E+06 b	4.48E+07	4.95E+08	4.70E+07 a	1.84E+05 b	1.42E+05
Sign. trait	**	**	ns	ns	**	*	ns
LSD_0.05_ (n = 35)	1.17E+05	1.96E+06	1.12E+07	9.30E+07	8.26E+05	6.31E+05	2.86E+05

*NK* Without phosphate or compost addition, *COMP* Sorghum straw-based compost, *P-COMP* Sorghum straw-based compost + BPR, *P-COMP-SOIL* Compost made from sorghum straw, BPR, and sorghum rhizosphere soil, *BPR* Burkina phosphate rock, parameter values assigned by different letters differ significantly (**) at *p* < 0.01 and significantly (*) at *p* < 0.05, *ns* Not significant at *p* < 0.05. *SE* Standard error, *error df* Error degree of freedom, *DMRT* Duncan’s multiple range test, *LSD* Least significant difference, *phnX* Phosphatase, *phoD* Alkaline phosphatase, *ITS* Internal transcribed spacer (total fungi), *16S rRNA* (total bacteria), *entA* Enterobactin-mediated siderophore, *pqqE* Pyrroloquinoline quinone, *aphA* Acid phosphatase*,* *DAS* Days after sowing, [*S5, S8, S9*] Stage 5 (boot), stage 8 (hard dough), and stage 9 (physiological maturity) of the sorghum variety *kapelga.*

Table 5 also shows a highly significant effect (*p* < 0.01) of sampling period on *phnX, phoD,* and *entA* abundances, and a significant effect (*p* < 0.05) on *pqqE* in soil*.* The overall abundance of rhizosphere soil *phnX* increased with progress in cultivation and was significantly higher at harvest (5.18 10^5^ copies g^−1^ of dry soil). A similar trend was observed for *entA*, although the overall abundance at 115 DAS was not significantly different from that at 52 DAS. In contrast, the *phoD* and *pqqE* genes were significantly more abundant in the soil at 52 DAS than at 93 and 115 DAS.

### Multivariate analysis: interactions between soil chemical and microbiological properties and sorghum yields

**Figure 1:**
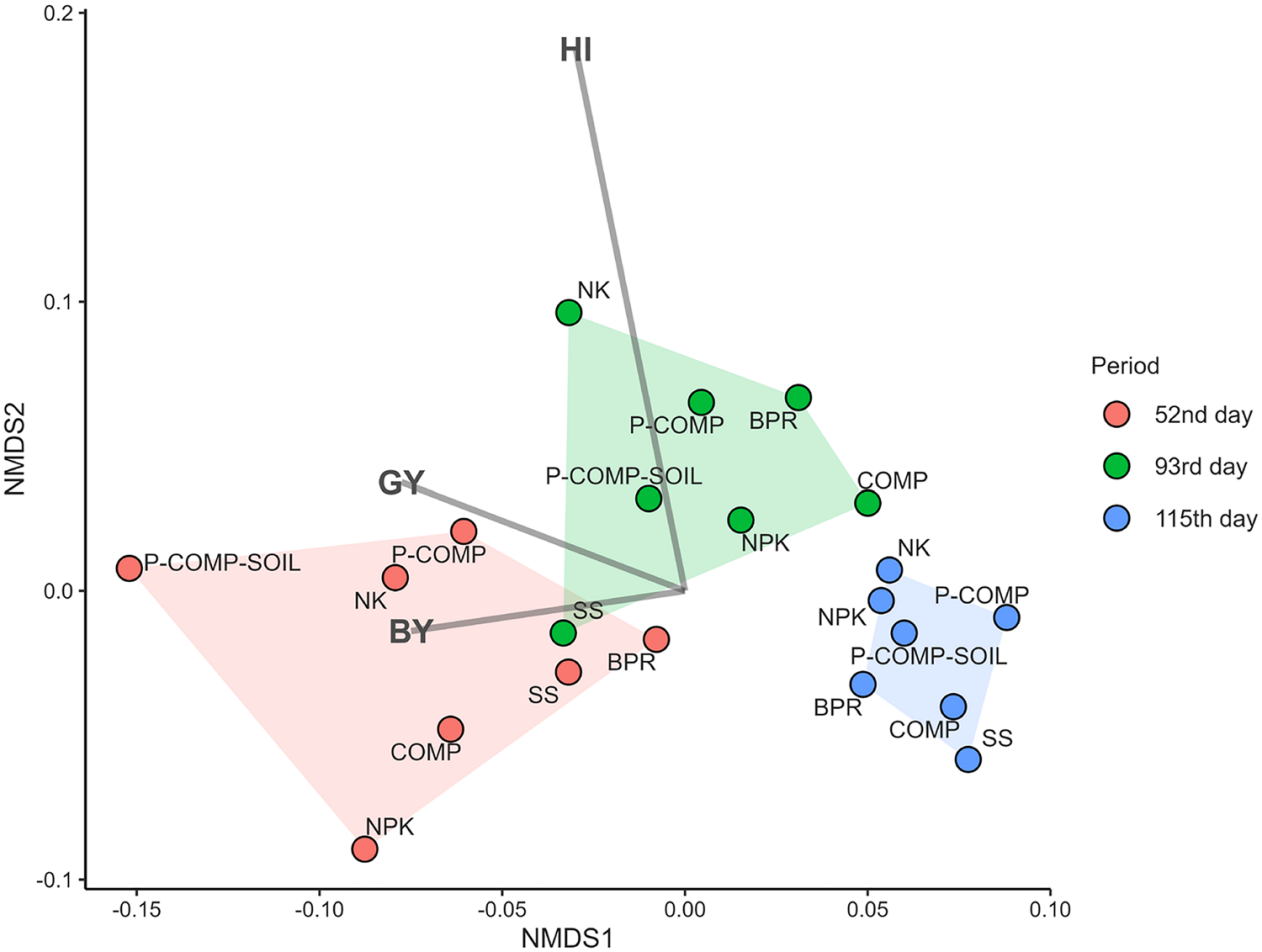
Nonmetric multidimensional scaling (NMDS) of sorghum yield components and soil characteristics. BY = biomass yield, GY = grain yield, NK = control without phosphate or compost addition, SS = sorghum straw, COMP = sorghum straw-based compost, P-COMP = sorghum straw-based compost + BPR, P-COMP-SOIL = compost made from sorghum straw, BPR and sorghum rhizosphere soil, BPR = Burkina phosphate rock. Vectors exert more influence on a Non-metric Dimensional Scale (NMDS) if they are located further away from the NMDS origin. A narrow angle implies a positive correlation between two variables, whereas a large angle suggests a negative correlation. A 90° angle indicates no correlation between the two characteristics.

NMDS was performed to investigate the relationships between soil properties during the three sampling periods and sorghum yield components (Fig. 1). The sampling period significantly influenced the parameters investigated in the present study. For instance, the earlier growth stages (52 DAS and 93 DAS) significantly influenced sorghum yield components more than the harvest period. We observed that P-COMP-SOIL and NPK at 52 DAS resulted in better sorghum yields than the other five treatments, which was not the case at 115 DAS (harvest).

Spearman’s correlation (Table S4) confirmed that sorghum biomass was moderately negatively correlated with *entA*, AMF, pH, and Bray2_P, and moderately positively correlated with TN, TC, and exchangeable cations. Grain yield was moderately negatively correlated with AMF, pH, and Bray2_P, and positively correlated with the abundance of bacteria (*16S rRNA*), TN, TC, and exchangeable cations. Soil bacteria abundance was moderately positively correlated with AMF, pH, and exchangeable cations, but weakly correlated with C/N. Soil fungi were moderately negatively correlated with *pqqE* and weakly negatively correlated with N and C. In addition, it correlated moderately positively with AMF, weakly with pH, and strongly with bacteria. The *phnX* gene was strongly positively correlated with AMF and pH, moderately positively correlated with total fungi and total bacteria, and moderately negatively correlated with *phoD* and *pqqE*.

## Discussion

The excessive use of fertilizers in many parts of the globe, especially N and P fertilizers, accelerates environmental change and hampers human sustainable development^41^. However, the two elements are distributed unevenly in soils globally. They are insufficient in most weathered tropical soils, especially in sub-Saharan Africa, resulting in low crop production. Many studies have revealed that total P might be present in the soil at much higher levels than required for plant growth; however, the soluble fraction available to plants is small^42,43^. In the study area in the present study, 319.8 mg total P kg^−1^ soil^30^, 5.78 mg Bray1-P kg^−1^ soil, and 5.28 mg Bray2-P kg^−1^ soil could be recovered. The critical P threshold of 11.6 mg Bray1-P kg^−1^ soil required for sorghum growth in West Africa^44^ justifies the need for P fertilization. In the present study, excluding the NK, all treatments supplied the same amount of total P (39 kg ha^−1^). However, the P sources differed and were either TSP (NPK treatment), entirely from BPR (BPR treatment), or from the mixture of organic material and BPR (Sorghum straw, COMP, P-COMP, and P-COMP-SOIL treatments), as shown in Table S2.

The higher amount of available P supplied by TSP (NPK treatment) and P-COMP-SOIL could explain the yield difference observed between the two treatments and the rest of the treatments. Sorghum biomass and grain yields in the NK were 34 and 27% lower, respectively, than those in the NPK treatment, highlighting the importance of P nutrition in the studied environment, further confirming that P is a limiting nutrient in the soil. Many studies have highlighted the significant contribution of improved P uptake to crop grain yields in Africa^45,46^. In addition, we observed 25 and 23% decreases in biomass and grain yield, respectively, in the NK compared to in the P-COMP-SOIL. P-COMP-SOIL appears to be a suitable organo-mineral fertilizer alternative to the more expensive chemical fertilizer NPK, supplying adequate plant-available P and higher levels of exchangeable cations (Table 2) necessary for plant growth. The organic material in compost treatments improves soil physical, chemical, and biological health^47^. It acts as a glue holding soil mineral matter together, thus influencing soil structure and associated properties, such as soil water storage and supply capacity. Biologically, it supplies nutrients and energy required by plants and associated microorganisms for metabolic activities. Plants further stimulate microbial activity by providing microorganisms with an energy source, typically C compounds. Interestingly, P-COMP-SOIL had a significantly higher percentage of good panicles (filled with grains), similar to the NPK, COMP, and P-COMP treatments.

Although we applied higher N, P, and K rates throughout the different treatments, the sorghum yields obtained in the present study were low. However, they were twice as high as those from other studies^30^ conducted in the same area during the same season. In these previous studies, the authors achieved an average sorghum grain yield of only 0.34 t ha^−1^ in 2019, while the grain yield in 2018 was 1.47 t ha^−1^, when the rates of application of N, P, and K were 37, 10, 9 kg ha^−1^, respectively, according to the blanket recommendation in Burkina Faso^48^. The low amounts of available soil water in 2019 compared to 2018, especially in the early days of cultivation^30^, negatively affected the establishment of sorghum seedlings, which translated into low yields, in the present study. However, the two highest sorghum yields obtained after N, P, and K supplementation at higher rates indicated that the elements are still limiting in the area, and the blanket recommendations made in 1978 should be reconsidered. Most often, national fertilizer recommendations ignore intra-regional variations in soil fertility.

Bray2-P was higher in the sorghum rhizosphere soil than in the bulk soil, indicating a significant rhizosphere effect on P solubilization/mobilization. The rhizosphere effect refers to the cascade of molecular events at this specific plant-soil interface, which governs microbial metabolism and plant nutrition^49^. Several plants and microbial compounds, such as organic acids, phytohormones, and P-solubilizing/mineralizing enzymes that are released in the area^15,50^ are crucial for biogeochemical transformations. Indeed, the significant decrease in Bray2-P in bare soil when no plant was grown following TSP addition (Fig. S3) is considered to result from P adsorption to the soil in the absence of the rhizosphere solubilization effect. Unfortunately, the total soil P with TSP addition and after was not quantified to confirm the P adsorption levels in the soil. However, most of the P applied to the soil as mineral fertilizers or organic manure is bound to the soil, augmenting the residual P pool, and other parts are lost by leaching and runoff^51^. Therefore, enhancing P solubilization, particularly in the rhizosphere of plants, is essential for increasing P-use efficiency and crop production. In the present study, in addition to supplying higher levels of labile P to the soil than the BPR and the organic material treatments, the P-COMP-SOIL fertilizer enriched the soil microbiome, including P-solubilizers, whose activity could be further promoted by the rhizosphere effect via exudates of molecular compounds from sorghum roots. Indeed, C compounds released from plants through rhizodeposition are a vital source of nutrients for soil microbes^52^ that exhibit increased development with an increased release of the compound and other phytochemicals in the exudates. The total bacterial abundance was significant in the rhizosphere of P-COMP-SOIL treatment from the early growth stage (52 DAS). The treatment also showed a significantly high presence of the organic phosphate-mineralizing gene, *aphA*, together with the COMP and NPK treatments, and the *phnX* gene, together with the COMP and P-COMP treatments. Most importantly, the expression of the primary inorganic P-solubilizing gene *gcd* in the rhizosphere was significantly higher under the P-COMP-SOIL treatment, at 52 DAS. Such genes may be expressed at the beginning of the growth period for the early mobilization of available P for sorghum growth. Early biogeochemical nutrient transformations are essential and provide plants with an array of available nutrients^53^ for their establishment, which significantly influences yield at harvest, as illustrated in the NMDS results (Fig. 1).

Notably, the available P content was relatively low at harvest in the P-COMP-SOIL-treated rhizosphere soils, similar to in the NPK and P-COMP treatments. Although we did not analyze biomass and grain P uptake, the higher sorghum production obtained in the NPK and P-COMP-SOIL treatments likely resulted in increased labile-P uptake, lowering its concentration in the rhizosphere. A significant positive correlation between sorghum yield and P uptake has been reported in the same area^30^. In addition, rhizobacterial species richness improves sorghum growth and nutrient synergism in nutrient-poor soils, and soil nutrient contents are generally lower under high plant-associated rhizobacterial diversity^54^. The significant abundance of the microbial *pstS* gene in the P-COMP-SOIL-treated rhizosphere soil may indicate better transport of available P in the treatment (Table 3). In bacteria, the phosphate import system is activated when the external phosphate concentration is ≥ 20 µM^55^. Arbuscular mycorrhizal fungi are another important symbiotic microbial group that improves plant nutrient uptake, especially P and N intake^56,57^. Their significant presence in the rhizosphere soil of the NK treatment without P application, and the P-COMP-SOIL and P-COMP treatment, at 52 DAS, indicate P limitation in the soil in a range that still allows AMF development to support sorghum growth. AMF reportedly improve the growth and production of wheat, rice, maize^58^, and sorghum^59,60^ in P-limited soils. The decrease in available P during the cultivation period in the P-COMP-SOIL and NPK soils partly explains the weak negative Spearman’s correlation between yield and available P (Bray-2 P), as shown in Table S4. The higher amounts of Bray-2 P in the BPR, Sorghum straw, and COMP treatments, especially at 93 DAS and 115 DAS (Table 3), showed that P was still high in the rhizosphere soil at harvest. However, P may be retained by sorption onto metal (Fe, Al) hydroxides, bound to organic matter compounds^61^, or immobilized in microbial cells to be solubilized and reused by the organisms. The strong positive Spearman correlations between yields and N and C (Table S4) indicated that N and C are essential elements for sorghum growth in the studied environment, in addition to P and exchangeable cations. They were moderately negatively correlated with soil pH, showing that soil pH tends to decrease with increasing amounts of N and C in the soil. Moreover, the moderate negative correlation between yield and soil pH confirmed that soil acidity influences sorghum production in the study area. The NPK treatment, which had the highest sorghum yields, led to the lowest pH of 5.15 and 5.46 at 52 DAS and 93 DAS, respectively (Table 3). Soil pH was one of the drivers of P utilization in the studied soil. It was positively correlated with *phnX*, total fungi, and total bacteria, indicating the increased activity of the microbes harboring the genes when the pH was slightly higher.

## Conclusion

The results of the present study highlight the importance of soil fertilization in supporting crop production in sub-Saharan Africa. Although NPK effectively improved the yield components of sorghum in the studied area (Burkina Faso), BPR-rhizosphere soil-enriched compost is an alternative low-cost organo-mineral fertilizer. The rhizosphere soil described in the present study was the soil volume still attached to sorghum roots after gently shaking the uprooted plant stand to remove loose soil. Using the method above, it is possible to collect 30 kg of fresh soil from 15 to 20 individual plants. In addition, the enriched compost hosts a high abundance of microbial genes involved in the solubilization/mineralization and mobilization of phosphate (*gcd*, *pqqE*, *phnX*, *aphA*, AMF, and *pstS*), which may have partly contributed to the observed growth improvement, especially when their activity is promoted around the plant roots by the rhizosphere effect. According to the results of the present study, in addition to P, N, C, and exchangeable cations are elements required for sorghum growth, and organic matter application remains vital for the improvement of soil fertility in sub-Saharan Africa. The direct application of low-grade phosphate rocks might not be adequate in upland cultivation systems where water is generally limited.

## Supplementary Information

Supplementary Information.

## Data Availability

The datasets generated during and/or analyzed during the current study are available from the corresponding author on reasonable request.
