# Supplementary material for: Amendment with Burkina Faso phosphate rock-enriched composts alters soil chemical properties and microbial structure, and enhances sorghum agronomic performance
Source: Sci Rep. 2022 Aug 17;12:13945. doi: 10.1038/s41598-022-18318-1 (PMC9386011; doi:10.1038/s41598-022-18318-1)
Supplement: Supplementary file 1 — Supplementary Information. [file 41598_2022_18318_MOESM1_ESM.docx]

**Table S1** Calculation of the amount of organic material input and quantities of Burkina Phosphate Rock (BPR) and urea required for adjusting nitrogen and phosphorus rates of application as desired.

| a. Determination of the required amount of organic material for field application, and calculation of required BPR amount for P adjustment | | | | | | | | | | |
| --- | --- | --- | --- | --- | --- | --- | --- | --- | --- | --- |
|  | Total P (g kg^-1^ DM) | Required P (kg ha^-1^) | Required dry Material (kg)† | % dry organic material (OM)†† | Required fresh material based of %OM (kg)††† | Applied fresh material  (t ha^-1^)†††† | Applied dry material (kg)‡ | Applied amount of P (kg ha^-1^)‡‡ | Required extra P  (kg ha^-1^)‡‡‡ | Adjustment with BPR  (kg ha^-1^)‡‡‡‡ |
| Sorghum straw | 0.286 | 39 | 137308 | 94 | 146072 | **1.34** | 1254 | 0.36 | 38.91 | 314 |
| Compost | 1.319 | 39 | 29780 | 68 | 43795 | **1.34** | 907 | 1.20 | 38.07 | 307 |
| P-compost | 60.061 | 39 | 654 | 49 | 1335 | **1.34** | 654 | 39.00 | 0.00 | 0 |
| P-compost-soil | 67.246 | 39 | 584 | 38 | 1537 | **1.34** | 507 | 34.10 | 5.17 | 42 |
| b. Calculation of required urea amount for N adjustment | | | | | | | | | | |
|  | Total N (g kg^-1^ DM) | Required N (kg ha^-1^) |  |  |  | Applied fresh material  (t ha^-1^)†††† | Applied dry material (kg)‡ | Applied amount of N (kg ha^-1^)‡‡ | Required extra N  (kg ha^-1^)‡‡‡ | Adjustment with urea  (kg ha^-1^)‡‡‡‡ |
| Sorghum straw | 2.755 | 60 |  |  |  | **1.34** | 1254 | 3.46 | 56.54 | 123 |
| Compost | 21.465 | 60 |  |  |  | **1.34** | 907 | 19.48 | 40.52 | 88 |
| P-compost | 27.068 | 60 |  |  |  | **1.34** | 654 | 17.70 | 42.30 | 92 |
| P-compost-soil | 29.069 | 60 |  |  |  | **1.34** | 507 | 14.74 | 45.26 | 98 |

DM = dry material, BPR = Burkina Phosphate Rock, † = Required dry material to cover 39 kg P ha^-1^, †† = calculated after determining the moisture content in materials, ††† = Required amount of fresh organic material, †††† = corresponds to the lowest amount of required fresh material (that of P-Compost, **1.34 t ha^-1^**), ‡ = corresponds to the exact amount of dry material out of the applied 1.34 t ha^-1^ fresh material, ‡‡ = applied amount of P (g P kg^-1^ DM * applied dry material) and applied amount of N (g N kg^-1^ DM * applied dry material), ‡‡‡ ­= amount of extra P to reach the required 39 kg P ha^-1^ and amount of extra N to reach 60 kg ha^-1^, ‡‡‡‡ = amount of supplemental BPR and urea to fill the required P and N application rates, considering that BPR contains 12.4% P and urea contains 46% N.

**Table S2** Summary of the amounts of applied organic material, Burkina Phosphate Rock (BPR), urea, triple super phosphate (TSP), and potassium chloride KCl in treatments

|  | Sorghum straw | Compost | P-compost | P-comp-soil |  | BPR | Urea | TSP | KCl | (%)P [OM-BPR/TSP] |
| --- | --- | --- | --- | --- | --- | --- | --- | --- | --- | --- |
| Treatments | t ha^-1^ | | | |  | kg ha^-1^ | | | |  |
| Control (+N, -P, +K) | 0 | 0 | 0 | 0 |  | 0 | 130 | 0 | 48 |  |
| Sorghum straw + BPR | 1.34 | 0 | 0 | 0 |  | 314 | 123 | 0 | 48 | [1.11-98.89] |
| Compost + BPR | 0 | 1.34 | 0 | 0 |  | 307 | 88 | 0 | 48 | [3.33-96.67] |
| P-compost | 0 | 0 | 1.34 | 0 |  | 0 | 92 | 0 | 48 | [100-0] |
| P-compost-soil + BPR | 0 | 0 | 0 | 1.34 |  | 42 | 98 | 0 | 48 | [86.67-12.33] |
| BPR | 0 | 0 | 0 | 0 |  | 315 | 130 | 0 | 48 | [0-100] |
| NPK (60-39-25) | 0 | 0 | 0 | 0 |  | 0 | 130 | 196 | 48 | [0-100] |

OM = organic material, BPR = Burkina Phosphate Rock, TSP = Triple Super Phosphate, P-Comp = sorghum straw-based compost + BPR, P-Comp-soil = compost made from sorghum straw, BPR and sorghum rhizosphere soil, 1.34 t ha^-1^ is determined as in Table S1 (any rate higher than 1.34 t ha^-1^ would supply more than the required amount of phosphorus [P] in P-Comp), the amounts of BPR and urea are from the last column of Table S6 in addition to that of Control, BPR and NPK treatments that received nitrogen (N) entirely from urea and BPR treatment that received its P entirely from BPR, 196 kg TSP ha^-1^ is needed to supply 39 kg P ha^-1^, and 48 kg KCl ha^-1^ is needed to supply 25 kg K ha^-1^ (KCl is uniformly applied to all treatments), the last column shows the proportions of P supplied by P sources (organic material-BPR or TSP).

**Table S3** Amplification efficiencies and r^2^ values of microbial genes determined using qPCR

| Microbial genes | Amplification efficiency ranges (%) | r^2^ values |
| --- | --- | --- |
| 16S rRNA (bacteria) | [81.4-93.9] | [0.995-0.999] |
| ITS (fungi) | [79.5-83.3] | [0.999-1.000] |
| AMF | [76.7-87.0] | [0.998-0.999] |
| *gcd* | [83.2-84.5] | [0.997-0.999] |
| *pqqE* | [95.2-102.2] | [0.997-0.999] |
| *aphA* | [83.7-93.6] | [0.996-0.999] |
| *phoD* | [57.2-60.0] | [0.999-0.999] |
| *phnX* | [75.0-94.5] | [0.996-0.999] |
| *entA* | [91.7-102.1] | [0.997-0.999] |
| *pstS* | [73.5-76.2] | [0.996-0.999] |

**Table S4** Spearman’s correlation (rs) between soil physicochemical and biological properties, and sorghum yield.

|  | *phnX* | *phoD* | Tot-Fun | 16S | *entA* | AMF | *pqqE* | pH | TN | TC | C/N | Exch._  cat. | Bray2_P | DBY |
| --- | --- | --- | --- | --- | --- | --- | --- | --- | --- | --- | --- | --- | --- | --- |
| *phoD* | **-0.46***** |  |  |  |  |  |  |  |  |  |  |  |  |  |
| Tot-Fun | **0.54***** | **-0.28*** |  |  |  |  |  |  |  |  |  |  |  |  |
| 16S | **0.40**** | 0.04 | **0.60***** |  |  |  |  |  |  |  |  |  |  |  |
| *entA* | **0.33*** | -0.01 | 0.13 | 0.19 |  |  |  |  |  |  |  |  |  |  |
| AMF | **0.62***** | **-0.33**** | **0.40**** | **0.53***** | **0.42***** |  |  |  |  |  |  |  |  |  |
| *pqqE* | **-0.43**** | **0.53***** | **-0.43**** | -0.01 | 0.03 | **-0.41***** |  |  |  |  |  |  |  |  |
| pH | **0.65***** | **-0.33**** | **0.38***** | **0.41***** | **0.25*** | **0.51***** |  |  |  |  |  |  |  |  |
| TN | **-0.32**** | **0.45***** | **-0.34**** | 0.03 | -0.18 | **-0.23*** | **0.43***** | **-0.52***** |  |  |  |  |  |  |
| TC | -0.13 | **0.39***** | **-0.27*** | 0.13 | -0.03 | -0.03 | **0.30*** | **-0.35***** | **0.91***** |  |  |  |  |  |
| C/N | **0.38***** | 0.02 | 0.15 | **0.32**** | **0.38***** | **0.44***** | -0.14 | **0.26*** | 0.05 | **0.43***** |  |  |  |  |
| Exch._cat. | 0.12 | 0.21 | 0.02 | **0.39***** | 0.11 | **0.27*** | 0.07 | 0.04 | **0.53***** | **0.63***** | **0.38***** |  |  |  |
| Bray-2_P | 0.20 | -0.07 | 0.21 | -0.14 | 0.11 | 0.10 | -0.18 | 0.18 | -0.14 | -0.06 | 0.17 | 0.04 |  |  |
| DBY | -0.14 | -0.13 | 0.08 | 0.39 | **-0.47*** | **-0.43*** | 0.16 | **-0.39*** | **0.52**** | **0.51**** | 0.10 | **0.46*** | **-0.38*** |  |
| DGY | -0.27 | -0.16 | 0.10 | **0.45*** | -0.40 | **-0.49*** | 0.10 | **-0.43*** | **0.50**** | **0.49**** | 0.15 | **0.41*** | **-0.38*** | **0.94*** |

Except for the dry biomass yield (DBY) and dry grain yield (DGY) collected at harvest (115 days after sowing), all other parameters are data from the first, second, and third samplings (52-93-115 days after sowing). Significant Spearman’s (rs) correlations are in bold and preceded by the probability levels (****p* < 0.001, ***p* < 0.01, **p* < 0.5). The strength of the correlations is considered “very weak” if rs values range between “0.00 and 0.19,” “weak” between “0.20 and 0.39,” “moderate” between “0.40 and 0.59,” “strong” between “0.60 and 0.79,” and “very strong” between “0.80 and 1.00.” TN, total nitrogen; TC, total carbon; Exch. cat = Sum of exchangeable cations, Bray-2_P = available phosphorus by Bray-2 method, *phnX* = phosphonatase gene, *phoD* = alkaline phosphatase gene, Tot-Fun = Total Fungi (*ITS*), 16S = Total Bacteria (*16S rRNA*), *entA* = enterobactin gene (siderophore), AMF = arbuscular mycorrhizal fungi, *qppE* = pyrroloquinoline quinone E. *gcd*, *aphA*, and *pstS* were not included in the table since they all showed no significant interaction with any of the variables.

**Figure S1** pH dynamics in bulk and rhizosphere soils during sorghum cultivation

DAP, days after planting; Control, without phosphate or compost addition; SS, sorghum straw; Comp, sorghum straw-based compost; P-Comp, sorghum straw-based compost + BPR; P-Comp-soil = compost made from sorghum straw, BPR, and sorghum rhizosphere soil; BPR, Burkina phosphate rock; B, bulk; R, rhizosphere. For bulk soil, the pH was determined at 115DAP only where it did not significantly differ between treatments, DAS = days after sowing, [S5, S8, S9] = stage 5 (boot), stage 8 (hard dough), and stage 9 (physiological maturity) of the sorghum variety *kapelga*.

**Figure S2** Bray2-P dynamics in bulk and rhizosphere soils during sorghum cultivation

DAP, days after planting; Control, without phosphate or compost addition; SS, sorghum straw; Comp, sorghum straw-based compost; P-Comp, sorghum straw-based compost + BPR; P-Comp-Soil = compost made from sorghum straw, BPR, and sorghum rhizosphere soil, BPR = Burkina phosphate rock, B = bulk, R = rhizosphere, DAS = days after sowing, [S5, S8, S9] = stage 5 (boot), stage 8 (hard dough), and stage 9 (physiological maturity) of the sorghum variety *kapelga*.

**Figure S3** Available phosphorus in bare soil (0–10 cm) and at and after triple super phosphate (TSP) fertilization

Fert = fertilization, 0 d = bare soil was collected immediately after fertilization and analyzed for Bray2-P; 4 months = bare soil was collected after four months
